# The Relative Age Effect and Its Influence on Academic Performance

**DOI:** 10.1371/journal.pone.0141895

**Published:** 2015-10-30

**Authors:** Juan-José Navarro, Javier García-Rubio, Pedro R. Olivares

**Affiliations:** 1 Instituto de Estudios en Ciencias de la Educación, Universidad Autónoma de Chile, Santiago de Chile, Chile; 2 Individual differences, Language and Cognition LAB, Facultad de Psicología, Universidad de Sevilla, Sevilla, España; 3 Instituto de Actividad Física y Salud, Universidad Autónoma de Chile, Santiago de Chile, Chile; Örebro University, SWEDEN

## Abstract

**Introduction and Purpose:**

The policy of school organisation for grouping students in the same academic year is based on date of birth. The differences in the experiences and maturation of older students involve a relatively better performance in academic settings, which is known as the relative age effect (RAE). This effect is more important the younger the student is. The goal of this study is to identify the connections of influence that RAE, socioeconomic status (SES), and type of institution have on academic performance in a school population of eighth graders.

**Methods:**

The study is based on a population-based, representative sample of 15,234 8th graders (50.4% female; average age = 13.61 years) in the 2011 National System of Quality Assessment in Education Survey (SIMCE) from Chile. The SIMCE for global academic performance consists of 4 tests: reading, mathematics, social studies, and science. All tests consist of multiple-choice and closed questions. In addition, in order to have the information of general academic performance, an extra variable expressing the average score of each student was created. Also, the SIMCE includes additional variables for the evaluation process such as SES or type of school. Students were assigned to one of five age groups in terms of date of birth (G1, G2, G3, G4, and G5), in which students belonging to G1 are the oldest and students belonging to G5 are the youngest.

**Results:**

The results achieved in the structural equation modelling indicate a good global fit. Individual relationships show significant effects of the three variables observed on academic performance, although SES received the highest values. The influence of RAE took place both in the full sample and sub-samples composed according to the SES and academic performance, showing higher values for students with lower scores. Although the influence of RAE decreases when SES is controlled, its effect is still significant and contributes to additionally explain the performance.

**Conclusions:**

The RAE remains, even with residual values, an explanatory factor in academic performance even in eighth graders. Since the RAE decreases as the influence of schooling increases, the potential adverse effects for some students would be placed in previous and initial moments of formal schooling. These findings may be useful into taking steps towards flexibilisation on age of entry in compulsory schooling. Moreover, the need to implement early, comprehensive evaluation systems which include aspects related to neurodevelopment in order to provide maximum information to parents and educators is also drawn.

## Introduction

The policy of school organisation for grouping students in the same academic year is based on date of birth. In general, students who are born within the same calendar year are grouped in the same course [1]. This measure seeks for students to have the minimum possible differences among them. In Spain, for example, students are grouped according to the calendar year from January 1 to December 31. In Britain, England, and Wales, students born between September 1 and August 31 of the following year are grouped in the same course. This generates differences of up to one year of age in children with the same chronological age in addition to differences in biological age due to different maturation rhythms of students. The differences in the experiences and maturation of older students involve a relatively better performance in academic settings, which is known as the relative age effect (RAE) [2,3].

Most published studies on RAE and study samples on academic performance come from Britain [4], Norway [5,6], Belgium [7], or the United States [8]. Regardless of the differences between these studies, such as age of the participants, different backgrounds, students with special educational needs, and different sample sizes, the trend is that younger students within the school year will face more difficulties than relatively older students. This effect is more important the younger the student is, but it reverses when reaching higher education [9].

Several studies have attempted to explain this phenomenon, although inconsistencies in their results have been found. Medical reasons have been hypothesised [8] in which children born in winter are more deficient in vitamin D and are subsequently discarded [10]; the total time of schooling [11] has been considered when some students spend more time in school than others, also with unequal results [12]. At the moment, the hypothesis that has proved to be more reasonable is the RAE [7]. The month of birth has been considered decisive in explaining school performance as well as the retention rates of students with specific learning difficulties [8]. The progressive development of neuropsychological functions, such as attention, perception, or memory, as well as those more related to process control and cognitive self-regulation, have established important differences, especially near the age of compulsory school entrance. These differences appear to be a key factor in relation to possible decisions concerning both entry into the school system and grade repeating or possible implementation of compensatory measures [13]. Among the possible repercussions RAE could bring, one we can mention is teachers expecting less of their younger students due to their worse academic and social performance because of their later development. In this sense, the effect of lower expectations from teachers towards those who initially reported a lower degree of maturity reinforces a worse performance by those mentioned [14]. In addition, explanations of teachers are usually performed at the average level of the class, making it more difficult for younger students to understand and, therefore, more learning opportunities are lost [7].

From different explanatory models, developmental psychology has tried to respond to the psychological changes that occur in the development process. On the one hand, the cognitive–evolutionary models based on studies the of Piaget have maintained a particular interest in “normative” changes that occur throughout the life cycle in relation to age or rather in connection with certain phases or evolutionary periods in which human development is usually divided. One of the reasons that could explain this relationship between age and psychological changes would be in maturation [15]. Among the characteristics that define the human species is birth itself, which comprises a high degree of structural and functional immaturity which progressively, and following a certain maturation calendar, gives way to a greater degree of maturity. In this sense, maturation seems to follow a more fixed and predictable sequence in the early stages of the life cycle, giving way later to other influences such as culture, the historical moment, or the social group they belong to. Thus emerges a description of development based on stages that determines the appearance of certain achievements in general but does not clearly specify a relationship with education or culture. Although the RAE is not specifically addressed, it is understood from these models that the individual differences within a stage would be given by differences in the rates of maturation together with those from different experiences or learning opportunities. However, some evidence questions this thesis. In this sense, the acquisition of the same general cognitive structure (e.g. the notion of classification) as a prototypical function of a certain stage does not result in the successful application of that structure at different specific activities, since large gaps can occur. Also, the process of constructing certain functions appears to be related to the cultural importance given to these same functions [16]. These critical observations of the cognitive-developmental model question the “maturing thesis” as the central engine of psychological change and development of psychological functions.

For its part, from historical–cultural psychology, development is understood as a result of the appropriation of the elements and unique context clues of the cultural reference in which the individual is inserted [17]. From this perspective, education becomes a factor of development, and different forms of cultural mediation become the psychological instruments through which this occurs [18]. Psychological changes arise from the acquisition of new forms of cultural mediation, which in turn allow us to interpret and interact with the world in a qualitatively different way. From this perspective, it is understood that the development of higher functions, among which would be the logical reasoning, reading, writing, arithmetic operations, or strategic memory, is fundamentally a *cultural* development. However, within these models, it is also assumed that the construction of higher functions would be sustained on the basis of neuropsychological development and, more specifically, on the development of the neocortical brain structures both from a phylogenetic perspective and in terms of ontogenesis [19,20].

In relation to socio-cultural factors, one of the most studied variables on education relative to academic performance is socioeconomic status (SES) [21]. Currently, SES includes factors such as the level of education of the mother and father, family income, and family structure. In the meta-analysis by Sirin [21] from studies published between 1990 and 2000, a strong impact of SES on student academic performance was found. In this line, it states that differences in the type of institution, with low and high SES, were very important in performance. Aspects such as materials, experience of teachers, or teacher-pupil ratio determines academic performance, which, in turn, is determined by the SES [22,23].

To our knowledge, there are no studies that interactively relate the RAE, SES, and academic performance in Latin America with a national representative sample. In Chile, the levels of socioeconomic stratification are particularly pronounced. They constitute a social and political reality that is continuously on the agenda of reforms to be undertaken by national and supranational political institutions [24]. The results of a study from the Market Research Companies Association (AIM) [25], which divides the population into five groups according to SES, show that 57% of families make up Groups 4 and 5, which comprise the denominated low socioeconomic level (37%) and extreme poverty (20%). Over two-thirds of these families have not finished school; most require public assistance or subsidies to cover basic needs like housing, health, and education. In Chile, the majority of low SES families have enrolled their children in public schools. These results show that public schools are set around a cultural-disadvantage core, making it very difficult for these students to progress. Thus, socioeconomic differences are particularly reflected in the results obtained by the students of the several social strata. In this sense, the results of the various educational diagnosis tests, national and international, [26,27] repeatedly demonstrate the close connection between the SES of the family and academic performance. In fact, a PISA report reveals that Chile, even being one of the most successful countries in Latin America, shows low results in comparison with OCDE countries. Chile occupied the 50th position in mathematics (of 65 countries), 44 in sciences, and 45 in language in 2012. Moreover, according to PISA, the 23% variance in achievement in mathematics is explained by SES. Despite that, results of pupils within the top 20% for performance in Chile are in line with the mean of OCDE countries, showing a need of improvement in school systems. Also, in Chile, in regards to the RAE, the educational system allows students in the same course to be up to 15 months apart in age differences, so the potentially negative effects of this variable could become greater.

Given the above, this study has the following objectives: First, identify whether we can find a significant RAE in Chilean eighth-grade students, its level of influence on performance, whether this occurs both in relation to the global academic performance, and the different, specific, academic domains evaluated. Secondly, identify whether the influence of RAE occurs unevenly depending on the level of performance shown by students, the SES, or type of institution. Finally, identify to what extent the RAE provides an additional significance on performance in relation to the influence exerted by the SES and the type of educational institution. In relation to the proposed objectives, as a working hypothesis, we expected to continue to find a significant RAE on academic performance, even in eighth graders, although, as we have seen, the RAE has been highlighted especially in younger students. Also, we expected that this RAE was greater in those low-performing students belonging to public institutions and low SES. Finally, we expected that the RAE would contribute to explain further academic performance, providing additional information regarding the SES and type of institution.

## Materials and Method

In order to contrast our hypothesis, a correlational research design based on causal models is proposed [28].

### Participants

This paper analyses a nationally representative sample of students from 8th grade in the Chilean education system. The *National System of Quality Assessment in Education* (SIMCE) in Chile performs screening tests for all students enrolled that year. The data collection was conducted in September 2011 with a final participation in the study of 15,234 students (50.4% female). The average age was 13.61 (SD = 0.49). The data are available upon request here: http://www.agenciaeducacion.cl/investigadores/bases-de-datosnacionales/ (accessed 15 Dec 2014). This evaluation was approved under the Chilean Law of Sports number 19.712, Article 5 [29]. Written informed consent was required from every school prior to testing by the Ministry of Education (MINEDUC), and each school was instructed to inform parents and students with a standardised script about the nature and importance of the tests, the date and time of the assessment, and how to prepare for the tests [29]. Participants in the present study were students who took the test for the specific academic domain of physical education, and they were the only ones whose birthdates were requested. All the pupil information was anonymised and de-identified prior to analysis.

### Procedure

The application of the SIMCE tests takes place in each school by specifically trained professionals who do not belong to the respective institution. The Chilean Ministry of Education offers the databases with these test results to educational centres and research teams. The application of these tests is collective and performed in several course levels in primary and secondary education. The school year in Chile begins in March and lasts until December. Students who have the required age to March 31 may enrol in the respective course level. The system also allows students who meet the age requirement up to June 30 to enrol in the same course. For this study, students who at the time of the evaluation had not repeated a class and were born between March 1997 and June 1998 were chosen.

### Instruments and variables

The SIMCE for global academic performance consists of 4 tests: reading, mathematics, social studies, and science. All tests consist of multiple-choice and closed questions with a total score of 400 points. In addition, in order to have the information of global academic performance, an extra variable expressing the average score of each student was created. The SIMCE includes additional variables for the evaluation process such as SES or type of school. As referred above, these variables have demonstrated a very significant effect in relation to academic performance in the Chilean context [26].

The proposed hypotheses established an endogenous and latent variable that could be defined as the global level of academic performance as a result of the different areas of knowledge that make up the curriculum. An academic-performance measurement is performed through the 4 tests mentioned above for the different areas. Moreover, the exogenous variables of the study can pursue them in the family’s SES, the type of institution attended by students, and the RAE. SES was calculated using the educational level of the father and mother, incomes (self-reported), and the vulnerability index (through the National Board of Student Aid and Scholarships “JUNAEB”). Through cluster analysis, five groups were established: a) low, b) medium-low, c) medium, d) medium-high, and e) high [26]. Also, in the educational system in Chile, we can find three types of schools: a) public schools, where schools receive a grant or aid from the government for each student; b) subsidised private schools, which receive the same government aid as public schools but also a private fee; c) private schools, which operate without public funding [30]. In relation to RAE, the sample was divided into 5 groups based on date of birth, one for each quarter of the academic year. In this study, the first group included children born from 1 March to 31 May (1997), the second group from 1 June to 31 August, the third group from 1 September to 30 November, the fourth group from 1 December to 28 February (1998), and finally, the last group included the younger students, born from 1 March to 31 May (1998) according to Ministry of Education regulations. Students were assigned to one of five age groups in terms of date of birth (G1, G2, G3, G4, and G5). Students belonging to the G1 are the oldest and students belonging to G5 the youngest.

### Data Analysis

Descriptive statistics were performed with means, standard deviations, mean standard errors, and confidence intervals to characterise the study sample according to the each of the tests and the global academic performance score. Likewise, to analyse the differences between groups, an ANOVA was conducted using global academic performance as a dependent variable and as factors of the RAE, SES, and type of institution. Tamhane setting was used for post hoc comparisons between the different sub-groups. Once the data were set and after the verification of the previous assumptions for normality [31] as well as the verification of compliance with the conditions of order and range (model identification), we proceeded to apply the asymptotically distribution-free estimation method [32] in order to contrast the different explanatory relations under the proposed structural equation modelling. In the same way, we previously proceeded to analyse the main correlations between model elements, verifying that all were significant (p < .001). We also proceeded to check the global and incremental fit goodness and the parsimony of the model following the analysis of the indicators commonly used. Subsequently, we applied the causal model proposed, selecting different subgroups according to the SES and type of institution, and also with regard to the academic performance. Finally, a hierarchical, multiple-regression analysis was also conducted with the aim of analysing the additional explanatory power of the RAE over the controlled academic performance of the SES and the type of educational institution. In the application of statistical analysis, the programs SPSS v21 and AMOS v20 (Inc., Chicago, IL, USA), were used. Statistical significance was set at p < .05.

## Results

Table 1 shows the descriptive statistics for the scores obtained in each of the academic-performance tests corresponding to the different tested areas in the SIMCE as well as the global academic-performance score. The maximum score that can be obtained is 400 points. Table 2 shows these descriptive statistics based on different subgroups referred to in each of the exogenous variables of the study: RAE, SES, and type of institution. The result of the ANOVA (one way) performed in order to compare possible differences among subgroups is also shown as well as post hoc contrasts to examine between which subgroups the differences occur.

**Table 1:** Descriptive statistics for each of the tests that compose the SIMCE evaluation and for the global academic-performance score.

SIMCE Variable	*N*	Mean	*SD*	*SE*	95% CI Lower limit	95% CI Upper limit
AP-MAT	15234	262.44	47.43	0.38	261.69	263.19
AP-LEC	15234	258.90	48.36	0.39	258.13	259.67
AP-SOC	15234	262.52	48.21	0.39	261.75	263.28
AP-NAT	15234	266.45	50.07	0.41	265.66	267.25
GAP	15234	262.58	42.75	0.35	261.90	263.26

*Note*: SD = Standard Deviation; SE = Standard Error of the mean; CI = Confidence Interval of the mean; AP-MAT, AP-LEC, AP-SOC and AP-NAT = Rating academic performance in Math, Reading, Social Studies and Nature Science; GAP = Global academic performance score.

**Table 2:** Descriptive statistics according to different sub-groups of variables: *Relative Age Effect*, *Socio-economic status*, and *Type of Institution*, ANOVA results (one way) and post hoc analysis (all the comparisons post hoc presented are statistical significant).

*Relative Age Effect (RAE)*									
SIMCEvariable	ANOVA*F* (*p*-value)	Subgroups	*N*	Mean	*SD*	*SE*	95% CI Lower limit	95% CI Upper limit	*Post hoc (Tamhane)*
GAP	*F* = 23.24 *p* < .001	G1	1523	262.55	45.83	1.17	260.25	264.85	1 < 2** > 5**
		G2	3929	267.09	43.19	0.69	265.74	268.44	2 > 1, 3, 4, 5 **
		G3	3827	263.36	43.03	0.70	261.99	264.72	3 < 2** > 4, 5**
		G4	3490	260.42	41.54	0.70	259.04	261.80	4 < 2, 3 ** > 5 *
		G5	2465	257.24	40.49	0.82	255.64	258.84	5 < 1, 2, 3, 4 **
*Socio-economic Status (SES)*									
GAP	*F* = 955.87 *p* < .001	Low	1529	240.08	35.59	0.94	238.25	241.92	1 < 2, 3, 4, 5 **
		M-Low	4658	246.63	38.83	0.57	245.52	247.75	2 > 1 < 3, 4, 5 **
		Medium	5481	262.63	38.73	0.52	261.60	263.65	3 > 2, 1 < 4, 5 **
		M-High	2265	285.63	38.01	0.80	284.06	287.19	4 > 3, 2, 1 < 5 **
		High	1301	305.76	36.13	1.00	303.79	307.72	5 > 4, 3, 2, 1 **
*Type of Institution*									
GAP	*F* = 1372.85*p* < .001	Public	6561	246.91	38.76	0.48	245.97	247.84	1 < 2, 3 **
		Subsidized	7517	269.41	40.38	0.47	268.50	270.32	2 > 1 < 3 **
		Private	1156	307.08	35.78	1.05	305.02	309.15	3 > 2, 1 **

*Note*: SD = Standard Deviation; SE = Standard Error of the mean; CI = Confidence Interval of the mean; GAP = Global academic performance score;

*p < .05;

**p < .01.

**Fig 1:**
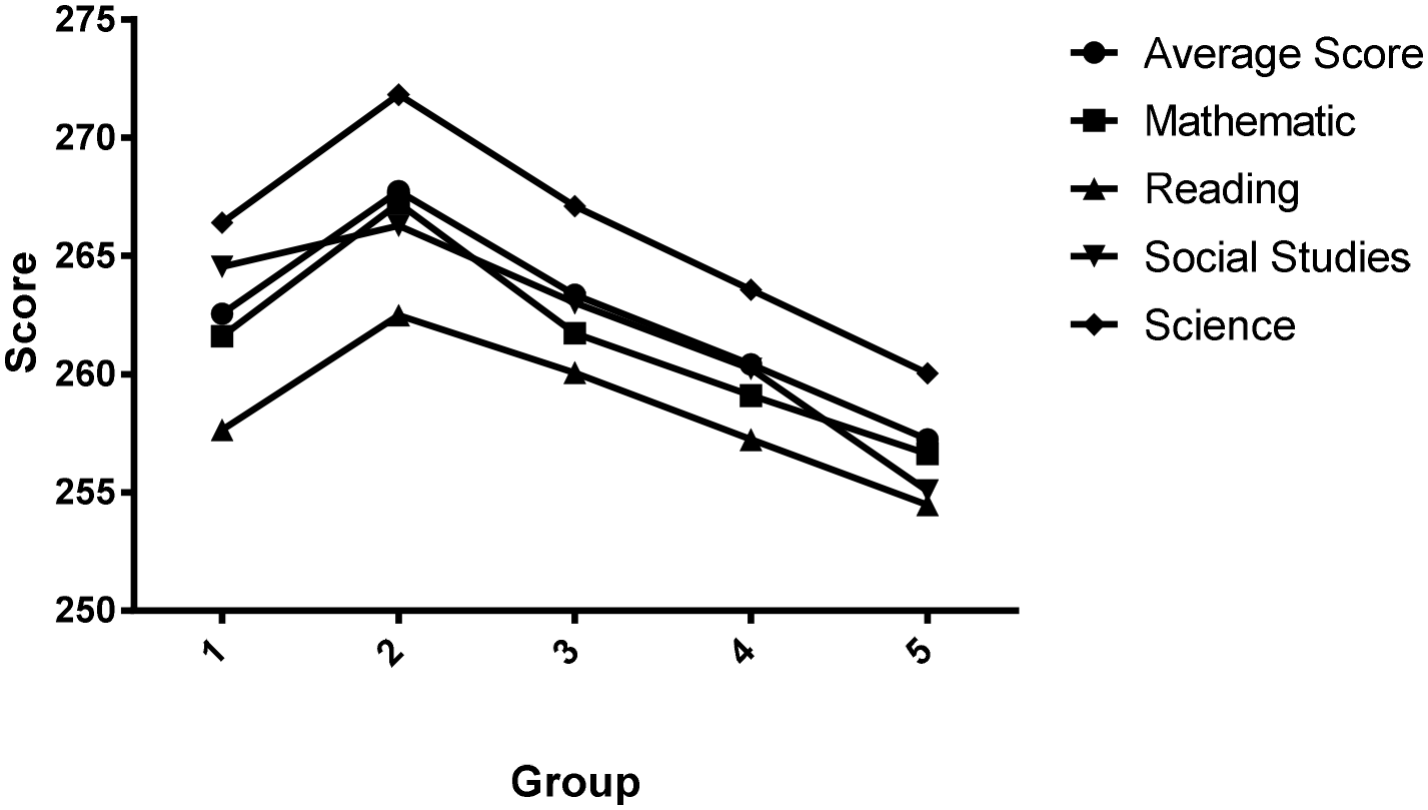
Average scores for each test according to birthdate.

The ANOVA showed significant effects of RAE, SES, and the type of institution on the global academic performance (average score) based on the specific tests of academic achievement. Regarding the RAE, the data show that G1 has lower score than G2; however, from G2 to G5 it shows a declining score, in line with the minor age of the students. Fig 1 graphically shows the trend observed in all measures.

Meanwhile, the post hoc analysis, made with test Tamhane without assuming equal variances, showed regarding the global academic performance that G2 established significant differences with the other groups. Likewise, G5 also established significant differences with the other groups. These results generally are repeated for each of the tests evaluated. In reading, however, this trend is not so clearly observed, and no significant differences among all subgroups with decreasing age were found; however, G2 obtained the highest score and established differences with three of the other groups. With regards to SES, results show significant differences among all groups, with higher scores on academic performance as SES increases. These results were replicated in each of the 4 tests evaluated. Meanwhile, in relation to the type of institution, results also reveal significant differences among the three groups referred to: private schools who obtain higher scores, followed by subsidised and, finally, public. As was the case for the SES, these results are replicated for each of the tests evaluated.

### Theoretical Structure of the Causal Model

**Fig 2:**
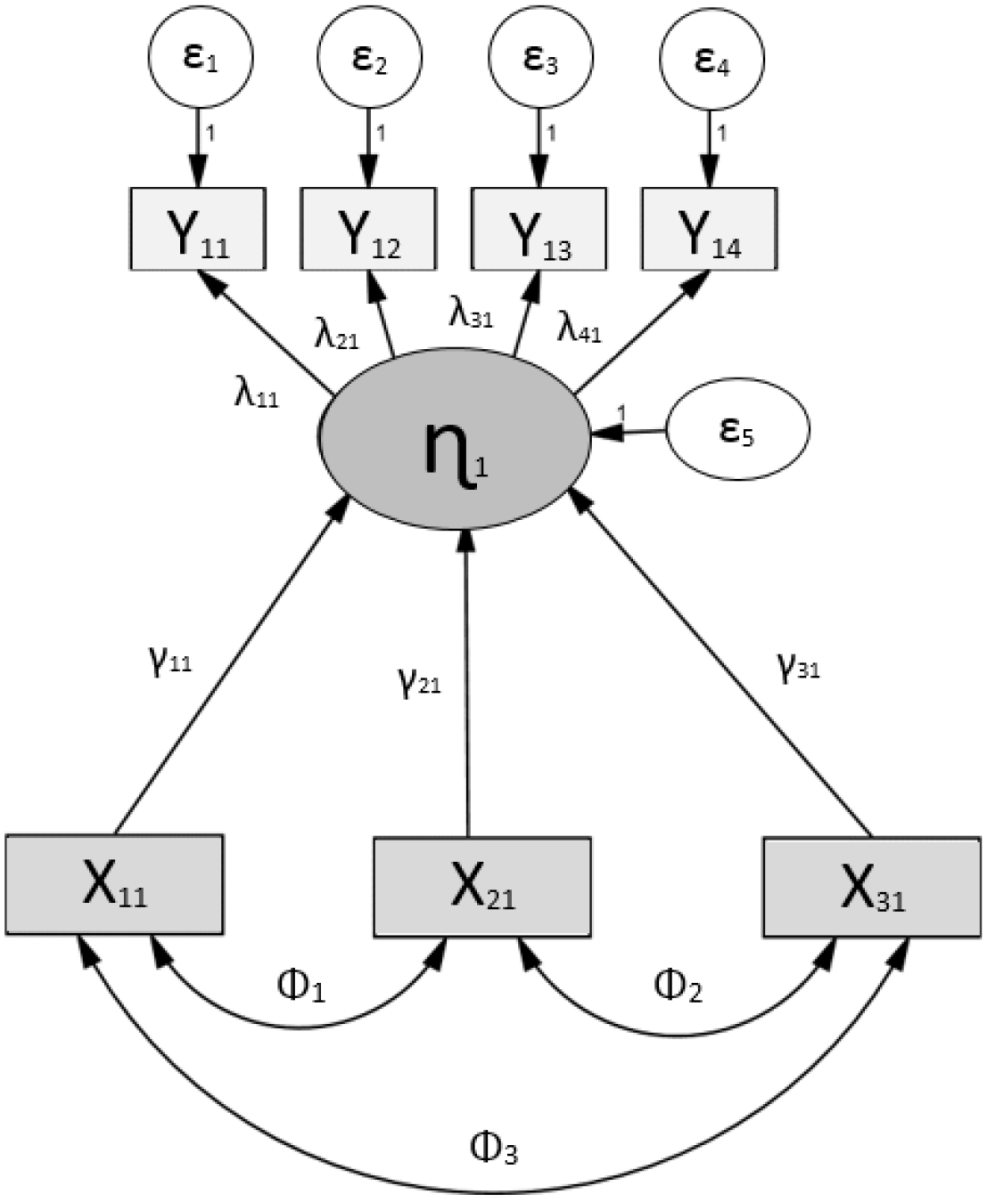
Design of theoretical structural equation modelling.

In relation to the objectives and hypothesis made as well as the conceptual review of the studied phenomenon, a theoretical causal model is proposed in order to contrast the extent to which it is consistent with the observed empirical data. This is a parsimonious model (Fig 2) which is used to see to what extent the global academic performance (ɳ_1_) latent variable measured from the 4 tests of academic achievement can be explained by the observed and exogenous variables that they have considered: the RAE (X_11_), socioeconomic status (X_21_), and type of institution (X_31_).

For its part, the observed variables that correspond to the dimensions that make up the global academic performance are formed from the scores on the tests of the specific areas of mathematics (Y_11_), reading (Y_12_), social sciences (Y_13_), and natural sciences (Y_14_).

### Contrast to previous assumptions to the analysis model

As a first step prior to analysis made with the structural equation modelling, several tests were made with the purpose of testing the assumptions of normality and identification of the model. First, regarding the normality assumption, the values of skewness and kurtosis of observed variables considered in the model were obtained (Table 3).

**Table 3:** Tests for normality for the variables included in the model.

Variable	Skew	C.R.	Kurtosis	C.R.
Relative Age Effect	0.016	0.830	-1.030	-25.960*
Socio-economic status	0.305	15.355*	-0.434	-10.947*
Type of Institution	0.405	20.393*	-0.667	-16.804*
Academic performance in Math	0.114	5.746*	-0.318	-8.023*
Academic performance in Reading	-0.144	-7.235*	-0.239	-6.025
Academic performance in Social Studies	0.150	7.571*	-0.618	-15.577*
Academic performance in Nature Science	-0.075	-3.801*	-0.485	-12.223*
*Multivariate*			-0.038	-0.207

*Note*: C.R. = Critical Ratio;

*α = .01.

The analysis shows positive skewness values in the variables of RAE, SES, type of institution, academic performance in math, and academic performance in social studies. Meanwhile, negative values of skewness are obtained in academic performance in reading and academic performance in natural science. Although these values are not significantly different from a symmetrical distribution, the test statistics obtained show lack of fit to a normal distribution (α = .01) in most cases. In this regard, it is good to remember that these statistics are particularly sensitive to sample size [31]. In regards to the kurtosis, negative values are obtained that indicate a platykurtic distribution with reduced concentration around the core values of the distribution. Test statistics also show in all cases’ values, which indicate a lack of fit to normality. It is estimated that critical values greater than 8.00 or less than -8.00 reflect a significant degree of non-normality. Meanwhile, the value of kurtosis-integrated multivariant and associated critical relationship also show that there is non-normality. Thereby, all the data obtained, together with the results of the test for normality Kolmogorov-Smirnov, in which no variable is set to normal (p < .001), lead us to conclude that this assumption is not fulfilled. In this sense, given that the sample size is greater than 1,000 subjects [33], it is decided to apply an asymptotically distribution-free estimation method [32].

In relation to the identification of the model, the analysis indicates that it is probably not a model identified. In this sense, statistical analysis, to achieve identifiability, imposed additional restrictions on the parameters. Moreover, the order condition was verified (11 degrees of freedom, corrected in function of non-identifiability) as well as the condition range (assuming that the covariance matrix is positively defined, the determinant of the covariance matrix is departs substantially from the value 0). Also, the lack of variance/covariance negative error, excessively high standard errors, or correlations between estimated coefficients above .80 was found.

### Checking of the model fit

When assessing model fit, which determines the degree to which the model predicts the observed covariance matrix, both absolute measures as incremental fit are taken into account. Also, measures of parsimony fit that provide information in relation to the simplicity of the model were entered. It should be noted that the application of nonparametric techniques for parameter estimation, especially with very large samples, have a lower efficiency than other parametric techniques [34], which could affect the scope of the proposed model.

In relation to the global model fit, Table 4 shows various indicators. First, the Chi-square contrast statistic shows a lack of global fit (p-value = .000). This result is expected based on the sensitivity of this statistical to sample size. In this sense, the Goodness of Fit Index (GFI), which is not affected by the sample size [31], shows a value indicative of good global fit (GFI = .995). The indicator of root mean square error of approximation also shows an acceptable value (RMSEA = .045).

**Table 4:** Indicators of model fit.

	Global Fit					Incremental Fit	Parsimony		
χ^2^	*d*.*f*.	*p*	GFI	CFI	RMSEA	AGFI	PRATIO	PCFI	*R* ^2^
355.265	11	.000	.995	.975	.045	.986	.524	.511	.23

*Note*: GFI = Goodness of Fit Index; CFI = Comparative Fit Index; RMSEA = Root Mean Square Error of Approximation; AGFI = Adjusted Goodness of Fit Index; PRATIO = Parsimony Ratio; PCFI = Parsimony fit to the CFI.

Regarding other indicators evaluated, the analysis relating to CFI index shows a value close to 1, indicating a good level of fit. For its part, the measure of incremental fit from adjusted GFI index is showing an optimum value of incremental fit (AGFI = .986). Likewise, parsimony-adjusted measures show values that are within the acceptable range (PRATIO = .524 and PCFI = .511). Finally, we can see that the model explains 23% of the variance of the latent variable.

### Analysis of individual relationships referred

**Fig 3:**
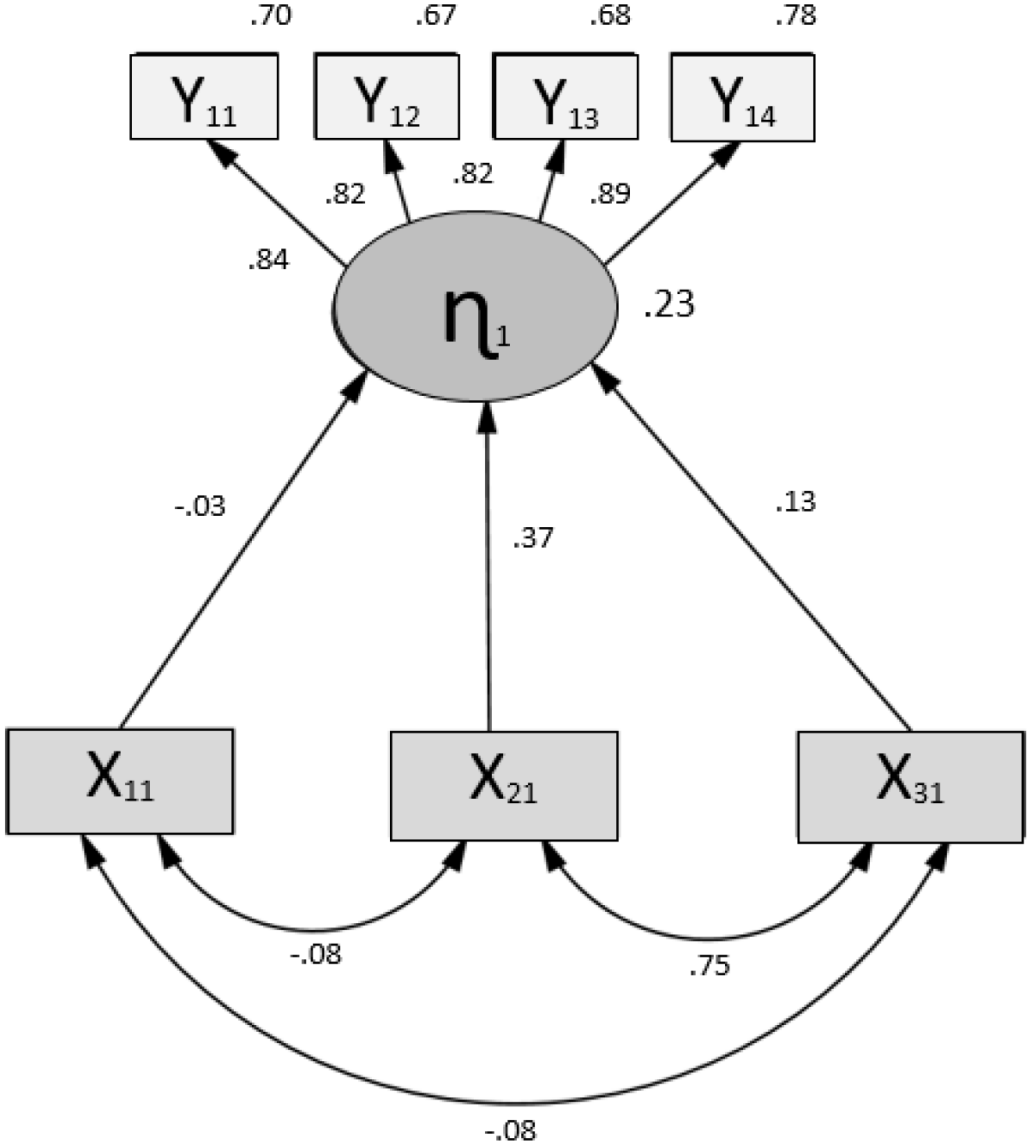
Standardised solution to the proposed model.

An individual analysis of the regression coefficients for each of the routes proposed in the model was made (Fig 3). In this sense, the standard solution of the model shows significant relationships between variables at a significance level of α = .01. In addition, both covariances and correlations between the exogenous variables are significant (p < .001). Table 5 shows the direct, indirect, and total standardised effects of the model.

**Table 5:** Direct, indirect, and total standardised effects between model variables.

	Effects	SES	INS	RAE	GAP
GAP	*Direct and Total Effects*	.367*	.135*	-.025*	.000
AP-MAT	*Direct*	.000	.000	.000	.838*
	*Indirect*	.307*	.113*	-.021*	.000
	*Total*	.307*	.113*	-.021*	.838*
AP-LEC	*Direct*	.000	.000	.000	.819*
	*Indirect*	.300*	.110*	-.021*	.000
	*Total*	.300*	.110*	-.021*	.819*
AP-SOC	*Direct*	.000	.000	.000	.822*
	*Indirect*	.301*	.111*	-.021*	.000
	*Total*	.301*	.111*	-.021*	.822*
AP-NAT	*Direct*	.000	.000	.000	.885*
	*Indirect*	.324*	.119*	-.023*	.000
	*Total*	.324*	.119*	-.023*	.885*

*Note*: AP-MAT, AP-LEC, AP-SOC and AP-NAT = Rating academic performance in Math, Reading, Social Studies and Nature Science; SES = Socioeconomic status; INS = Type of Institution; RAE = Relative Age Effect; GAP = Global Academic Performance.

*α = .01.

In relation to the observed exogenous variables, it is observed that the highest direct effect (.367) is produced by the SES (X_21_), while the RAE (X_11_) influences significantly even with a small effect (-.025). Likewise, the three exogenous variables indirectly influence (although with moderate or small effects) the 4 variables that make up the global academic performance, whose scores were obtained in tests of mathematics (Y_11_), reading (Y_12_), social sciences (Y_13_), and natural sciences (Y_14_).

On the other hand, based on the interest of this study to establish whether the RAE could have greater impact on certain groups of students in relation to SES or type of institution, the above analyses were performed selecting students who belonged, on one hand, to a low or medium-low SES who were schooled in public schools, and on the other hand, the high and medium-high SES schooled in private schools. Both cases contrasted the previous assumptions, same as was done for the entire sample. Depending on the characteristics of the sample and the analysis of normality index, the asymptotically distribution-free estimation method was applied [32]. The analysis indicates that there are probably unidentified models in both cases, winning two additional restrictions on the parameters to achieve identifiability. Order status (8 degrees of freedom, corrected for non-identifiability) and the rank condition were also checked, and a lack of variance/covariance negative error, excessively high standard errors, or correlations were found between the estimated coefficients above .80.

First, those students from lower socioeconomic groups (1 and 2) who were enrolled in the public school system were selected. This subsample consisted of 5,231 students, of which 1,334 formed Socioeconomic Group 1 and 3,897 who formed Socioeconomic Group 2. The results of the model application to this subgroup (which did not include the variable *type of institution*, since it had previously selected students who belonged to public schools) showed a direct standardised effect of RAE lower than on the full sample (-.01), while the effect of SES was .06. In relation to the global model fit on this subsample, the various indicators show an acceptable fit: χ^2^_(8, 5231)_ = 17.305 (p = .027), GFI = .999, AGFI = .998, CFI = .996, RMSA = .015, PRATIO = .533, PCFI = .531. The overall model explained only 0.4% of the variance.

The subsample composed of students of high and medium-high SES enrolled in private schools was comprised of 1,156 subjects, 20 of which formed the upper-middle socioeconomic group, and 1,136 of which formed the high socioeconomic group (Groups 4 and 5, respectively). The results showed a RAE standardised direct effect of -.02, while the effect of SES was .16. The global model fit indicators also showed an acceptable fit: χ^2^_(8, 1156)_ = 19.033 (p = .015), GFI = .993, AGFI = .981, CFI = .970, RMSA = .035, PRATIO = .533, PCFI = .517. In this case, the model explains only 3% of the variance.

Subsequently, analyses based on the level of performance obtained were performed in order to establish, on the one hand, whether the proposed model fits under the same conditions as student subgroups with low and high performance were analysed and on the other hand, to see whether the effects of observed variables remained equal. For this, the sample was divided according to academic performance average scores. It was established by a subgroup composed of students whose average performance score was below the 25th percentile (n = 3,808, Mean = 208.28, SD = 16.96). An additional group was established by another subgroup composed of students who scored above 74th percentile (n = 3,808, Mean = 318.45, SD = 18.88). Once this division was made, the analyses performed for the full sample were replicated for these subgroups of performance.

The results for students in the subgroup that scored below the 25th percentile (percentile < 25) showed a standardised direct effect of RAE = -.044, while the SES showed an effect of .081 and the type of institution, of .063. The global model fit over this subgroup shows acceptable indicators with the exceptions mentioned above relative to sample size: χ^2^_(11, 3808)_ = 57.550 (p = .000), GFI = .996, AGFI = .991, CFI = .958, RMSA = .033, PRATIO = .524, PCFI = .502. Despite these indicators, the model explains a whole 2% of the variance.

As for the subgroup of higher academic performance (percentile ≥ 75), the data showed RAE variable standardised direct effects similar to those obtained for the full sample (-.030). For its part, the parameters for the SES (.214) and type of institution (.145), despite being lower for the entire sample, were much higher than those shown for the subsample comprised of the lowest-performing students. As for the global model fit for this subgroup, the indicators generally show a good fit: χ^2^_(11, 3808)_ = 106.010 (p = .000), GFI = .993, AGFI = .982, CFI = .966, RMSA = .048, PRATIO = .524, PCFI = .506. In this case, the overall model explains 12% of the variance in performance.

Finally, in order to determine the additional predictive validity of RAE on academic performance, hierarchical multiple regression analyses were performed. In the regression analysis, the average score of academic performance obtained following the 4 tests evaluated was used as the dependent variable. Altogether, the variables’ values showed statistically significant correlation (p < .001), RAE being the only value that had a negative correlation with academic performance. The results of multiple regressions (Table 6) show how the variable SES has a significant impact on the academic performance, explaining 19% of it (Model 1), followed by the type of institution, which additionally explains 0.7% of the variance (Model 2). For its part, the RAE, after controlling the effects of SES and the institution type, shows a statistically significant effect on academic performance, additionally explaining only the 0.1% of the model (Model 3).

**Table 6:** Hierarchical multiple regression analysis for global academic-performance score on the Relative Age Effect, Socioeconomic status, and type of institution.

GAP (n = 15234)					
	Beta	*T*	*(p)*	*R*²	Δ*R*²
**Model 1**				.192	.192***
SES	.438	60.148	< .001		
**Model 2**				.199	.007***
SES	.345	31.179	< .001		
INS	.124	11.223	< .001		
**Model 3**				.199	.001***
SES	.344	31.101	< .001		
INS	.122	11.077	< .001		
RAE	-.025	-3.494	< .001		

*Note*: GAP = Global academic performance score; SES = Socioeconomic status; INS = Type of Institution; RAE = Relative Age Effect.

*** *p* < .001.

## Discussion

The objectives of this work aimed to identify whether RAE is present in Chilean eighth grade students, determining their degree of influence and whether it occurs in the different performance measures observed. Moreover, the study sought to analyse whether the possible influence of RAE took place in the same way when the sample was divided according to the different levels of SES, type of institution, and academic performance. Finally, the third objective refers to the analysis of the additional contributions of the RAE on performance in relation to the effects of SES variables and type of institution. In relation to these objectives, we set different working hypotheses. Regarding the first hypothesis, the results confirm the existence of the RAE in the Chilean educational system, showing significant effects on global academic performance and also on each of the measures to the different, specific, academic domains [1]. Overall, this significant effect is accompanied by small values. The influence of SES and to a lesser extent, the type of institution, is, as expected, significantly higher than RAE, showing major effects on performance across the board.

Regarding the results of the RAE, one of the most striking is the reverse effect of G1. This group shows lower values than the G2, but from this group, the trend is that younger students scored lower. As noted above, in the Chilean educational system, the parents can decide whether their children, born in the first quarter, enter into their natural age level or higher. This possibility enables students who stand out during their preschool stage to enter primary education earlier. This phenomenon also shows that the older students of the academic year were not the ones who stood out and may even have a learning disability, which would explain the results of G1. Some theories state that the sooner the student enters the school system, the easier it will be for him or her to learn while others say students need to reach a certain maturity to learn more complex content [6]. It has been found that students who enter school at age 7 obtained higher scores than those entering at age 6 [35,36]. On the other hand, studies show evidence from various international contexts of the relevance of early schooling for learning and school success, especially in situations of risk and sociocultural disadvantage [37,38]. In the particular case of Chile, although the data show that older students are not earning a higher academic performance, probably because they were not in a better position to choose to enter the previous year, they do obtain better performance than those born a year later whose are in the same course.

Between the possible reasons presented in the introduction of this article to explain the phenomenon of RAE, references to the differences in the rates of neuropsychological development of children [8] are made along with the relevance that could cause the low expectations that teachers can develop based on academic results achieved by students in previous courses [7]. It is possible that these reasons, along with some of the specific circumstances of the Chilean context noted above, are behind the results obtained. In this sense, among these circumstances we must mention the one that refers to the flexibility of the age when first entering compulsory primary schooling based on their previous results in preschool. Although this measure may be beneficial for some students it is possible that students may enter primary education and find that when facing age differences, it might turn into a handicap for their optimal educational development.

As mentioned above from the cognitive-developmental models in developmental psychology, the existence of horizontal gaps within the same stage of development and the different rhythms of maturation could explain the RAE, although this concept is not referred to in these models. From this perspective, a line divided into stages of development, which would have a universal and unchanging rhythm of acquisition, is established. These stages determine the appearance of the different functions in individuals, including either basic or *natura*l functions or higher mental functions [18]. In this sense, the reverse effect found in subgroup G1 would be inconsistent with the idea of development as a result of the independent maturation of sociocultural factors. Conversely, these data support the conceptions of more interactionists of development [15].

Meanwhile, the interactionist models that are more sociocultural or historical-cultural define development in terms of construction and internalisation of higher functions as a result of the individual’s participation in social situations in the reference context. The development, therefore, is not only influenced by culture but also is constructed from social interactions that take place within a given culture. From this perspective, individual differences, especially in relation to the development of higher mental functions, such as reading, arithmetic, or logical reasoning, occur mainly due to the appropriation of psychological instruments that mediate between culture and the individual. This *cultural* line of development helps to normalise psychological functions acquired by the school population [18], namely, to reduce variability in the observed scores of these functions. Thereby, it is assumed that the natural line of development would result in a greater dispersion of the measures, while the cultural line of development would be accompanied by lower dispersion of scores and greater normalisation. School, in this sense, fulfilled a homogeniser or normalising function. From these models the “normal” distribution of psychological functions is not assumed in the population as a result of an immutable genetic variability. On the contrary, the systematic acquisition of higher mental functions in formal education substantially alters the normal curve of development. In this regard, standardised measurements that assume a normal distribution of the population would mix the analysis of natural functions and higher functions. In the same vein, Sternberg and Grigorenko [39] establish that standardised measurements would be mixing consolidated functions with the measuring of developing functions. From these models, ultimately, differences linked to RAE should be understood as a consequence of the interaction and the variability of the result of this mixture in the measurement of natural and higher functions.

Also, from an interactionist conception, it is assumed that development is the result of a dynamic interaction of neuropsychological maturation and cultural mediation influences. [20]. In this sense, the decrease of the RAE as it moves schooling would be related to learning and internalisation of higher mental functions. This dynamic interaction between learning and development assumes neuroplasticity as an essential defining characteristic of human brain evolution. Thus, the normalisation in the development/learning of these higher functions would decrease the initial variability linked to the development of functions related to the natural line of development [18]. The data obtained in our study so clearly illustrate the influence of factors related to culture such as SES or the type of educational institution. The effect of these variables is statistically significant, further showing a practical significance [40] which corresponds to the social relevance of these variables in explaining academic performance in the Chilean education system. Meanwhile, although the RAE values are small, the significance shown must be taken into account by assuming an interactionist conception and a developmental approach as a variable which influences in interaction with others.

Regarding our second hypothesis, we expected that the RAE was higher in low-performing students belonging to public institutions and low SES. In this sense, it could occur that the RAE accentuates the poor performance of students who get lower scores while strengthening the possibility that low expectations appear in the school context based on poor performance. Also, we wanted to know whether the SES or the type of institution established differences in the observed effects. As mentioned above, the effect values found in the analysis for the RAE were generally small. The SES and educational institution have greater influence on academic performance than the RAE with somewhat larger values of the effect. In other studies in the field of physical education and sports, the effect sizes found for RAE are equally small [41,42]. These studies suggest the heterogeneity of the sample decreases the effects of date of birth.

However, focussing on our study, the data show that when the effects of SES and type of institution are controlled, namely with decreasing heterogeneity, they also decrease RAE. Also, analyses based on the academic performance showed lower levels of RAE in the subsample with lower dispersion in their scores (percentile ≥ 75), that is, with lower heterogeneity. The comparison made by the Pearson coefficient of variation actually showed a lower value of this coefficient (Cv = .059) for the subsample that received higher scores, accompanied by a lower value of RAE (-.030). Meanwhile, the coefficient of variation for the subsample scored lowest (percentile < 25) was higher (Cv = .081), with a value of RAE also higher (-.044). Similarly, the RAE decreases as the age of the student increases, and even reverses [9]. A much larger effect has been found in first grade students [7] and gradually disappears by the age of 12 [43]. These data suggest in turn that the effect of sociocultural factors increase as schooling progresses. That is in line with the arguments above that the further normalisation of the school population as a result of schooling, and the acquisition of higher mental functions help to reduce the heterogeneity and variability in the measurements observed of natural psychological functions, which would be parallel with the decrease in the RAE. In this sense, the data obtained in part confirm our second hypothesis because the RAE showed higher value in students with lower scores. It seems that the greater heterogeneity between the scores obtained by these students would have been a key factor in explaining higher RAE. In addition, since the relationship between SES and academic performance is very significant, potential negative effects of RAE could actually accentuate in this subgroup of students.

Finally, regarding our third hypothesis, we hoped that the RAE contributed to explain academic performance by providing additional information with respect to SES and type of institution. In this sense, the results support the relevance of RAE in academic performance, offering additional information to those explained by variables SES and type of institution. Although the increase in the proportion of explained variance is minimal, we must emphasise the practical significance that this contribution could have, especially in the early years of schooling in the face of decisions on measures such as flexibility in the stage of initial entry into school or at different stages of the same or the implementation of measures to help support students.

### Limitations of the Study

Within the limitations of the study, it should be noted that this work, even using a large representative sample of Chile, only analyses senior students of primary schooling. Undoubtedly, the analysis of academic paths according to the date of birth throughout schooling could give more information on how the RAE evolves in students across the 8 years of primary education. These studies also allow assessment of whether the initial or preschool moments, differences linked to RAE, are increased in situations of cultural disadvantage when the school still has not fulfilled its normalising function by providing tools to develop higher functions. Although the cultural differences become even larger in this way, the dispersion of scores of development of natural functions could lead to higher RAE values. Also, these studies would allow assessment of the effect of teacher expectations on academic performance in relation to those younger students. On the other hand, regarding the methodology used, we note that the non-identifiability of the model and the consequent need to impose restrictions on the parameters greatly increases the complexity as well as the distance of the results obtained with the reality. Also, we have already mentioned that the application of nonparametric techniques for parameter estimation is less efficient for model analysis [34].

## Conclusions

The RAE is a relevant factor when deciding on the right moment of schooling age. This could be especially important when it comes to children with low performance in previous stages, such as in preschool stage, as well as students at risk or with sociocultural disadvantages. In this sense, it seems essential to implement comprehensive and effective early-warning systems that include individualised assessments of the various areas of development in order to obtain valuable information and thus recommend (or not) an early start of compulsory primary education. These evaluation systems must provide information based on skills perceived by educators [44] and also dynamic tests adapted to the preschool stages which will provide access to their full learning potential [45]. Data from our study suggest, showing residual values at the beginning of adolescence, consistent with data from previous research that RAE decreases as age increases and the normalising influence of formal schooling increases. Despite this decrease, the RAE is still significant in the tested eighth graders, suggesting that its relevance would have been higher in earlier stages. Thus, even though the practise or substantive significance of the RAE, which is derived from data obtained, is small, the significance and social relevance could be especially important in the initial stages of schooling. Moreover, this seems to be more important for those students who show a poor performance in achievement tests. In this sense, the relevance of RAE contributes to rethinking the flexibility measures adopted in some educational systems in relation to the time of entrance to the school system of the students in different educational stages. Depending on the potential adverse effects that this factor can have throughout schooling, it is essential that counsellors and educators who are aware of these effects inform parents by providing precise guidance on whether or not to anticipate entry to the school system. Thus, the flexibility of these measures should take into account this factor, contemplating changes to the policy of school clusters, which are based on a better understanding of development, which in turn can increase the chances of all students.
